# "The War's Effect on a London Eye Hospital"

**Published:** 1914-12-12

**Authors:** H. R. S. Druce

**Affiliations:** Secretary, Central London Ophthalmic Hospital.


### "The War's Effect on a London Eye Hospital."

*To the Editor of* The Hospital.

Sir,—Your issue of December 5 contains a paragraph concerning the Central London Ophthalmic Hospital, in which you ask us a question, and I beg to furnish you with the answer.

You state that the administrative expenditure remains practically the same whether the beds are closed or not; this might be the case in a hospital with a large number of beds; it is not so in our case, since by closing these beds we thereby close a floor, making a considerable saving in the nursing and cleaning staff, fuel, light, etc.

With regard to the latter part of the paragraph, the writer is ignorant of the facts. There are two military depôts and recruiting stations close to the hospital, and many men are attending daily from these and also from distant camps to have their sight tested to enable them to pass the medical officer. Moreover, this hospital was one of the first of the voluntary hospitals to offer beds to the War Office for the treatment of the sick and wounded, the committee and staff entirely defraying the cost of equipping two wards for this purpose, and, although the public have been informed of this work for the nation, we have not yet "roped in new and increased subscriptions." In justice to us I trust you will publish this letter.—Yours faithfully,

H. R. S. Druce, Secretary,
Central London Ophthalmic Hospital.

December 7, 1914.

[Unless the *dead charges* were reduced when the wards were closed the cost per occupied bed remaining must have been thereby increased. This hospital only contains twenty-six beds. How many were offered to the War Office? The last paragraph of this letter would seem to indicate that funds have resulted.—Ed. The Hospital.]

